# How to do indocyanine green fluorescence‐guided axillary sentinel lymph node biopsy in breast cancer: the sunrise effect

**DOI:** 10.1111/ans.70122

**Published:** 2025-04-03

**Authors:** Chu Luan Nguyen, Deepali Poels, Basilie Teoh, Jue Li Seah, Carlo Pulitano, Sanjay Kumar Warrier

**Affiliations:** ^1^ Department of Breast Surgery Chris O'Brien Lifehouse Camperdown New South Wales Australia; ^2^ Department of Surgery Royal Prince Alfred Hospital Camperdown New South Wales Australia; ^3^ Department of Surgery The University of Sydney Camperdown New South Wales Australia

## Abstract

Indocyanine green fluorescence‐guided axillary sentinel lymph node biopsy.

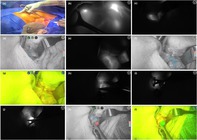

## Introduction

Sentinel lymph node (SLN) biopsy, performed using blue dye (BD) and technetium‐99 m lymphoscintigraphy, is the established standard for evaluating clinically node‐negative early breast cancer.^1^, ^2^ This technique achieves high SLN detection rates and low false‐negative rates of 96.7% and 5.5%, respectively.^3^ However, both BD and lymphoscintigraphy have disadvantages, prompting the exploration of alternative methods such as indocyanine green (ICG) fluorescence.^1^, ^2^

Indocyanine green fluorescence involves the use of ICG, a green fluorophore dye, in combination with a near‐infrared camera.^4^, ^5^ Upon binding to plasma albumin, the dye emits fluorescence when exposed to 806 nm of light, which is then captured on a near‐infrared display.^6^ Comparative cohort studies indicate that this technique offers results comparable to lymphoscintigraphy alone and superior to BD as a single technique.^7^ Despite growing evidence supporting its efficacy, the adoption of ICG fluorescence remains gradual due to the need for further high‐quality research and unresolved questions regarding cost‐effectiveness.^8^

This article outlines the surgical approach for axillary SLN biopsy in breast cancer using ICG fluorescence, focusing on key aspects of operative technique based on experience gained since its adoption in 2021 as a preferred alternative to BD.^8^, ^9^

## Surgical technique

### Administration

Patients undergo preoperative lymphoscintigraphy with peri‐tumour injection of technetium‐99 m. After administration of anesthesia and just before the procedure begins, 1 mL of Infracyanine® 25 mg/10 mL (SERB, Paris, France) is injected subdermally into the peri‐areolar region of the breast. A five‐minute manual massage facilitates lymphatic movement of the dye. The near‐infrared camera (SPY‐PHI, STRYKER, Sydney, Australia) can be affixed to the operating table, but handheld use is generally preferred for maneuverability.

### Indocyanine green fluorescence

**Fig. 1:**
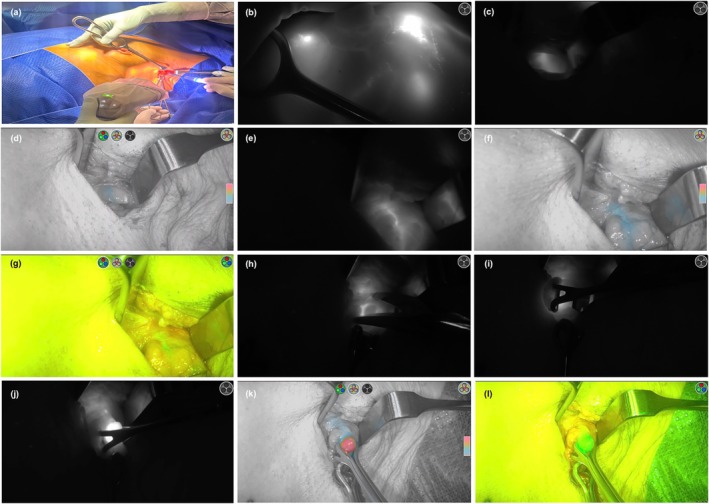
(a) Left axillary sentinel lymph node biopsy with near‐infrared camera setup. (b) Fluorescence of lymphatics observed following subdermal injection of ICG and massage. (c, d). Dissection through clavipectoral fascia and fluorescence identified within axilla on fluorescence mode and colour segmented fluorescence mode, respectively. (e–g) Axillary tissue retracted out with Babcock forcep demonstrating fluorescence of a lymphatic channel on fluorescence mode, colour segmented fluorescence mode, and overlay fluorescence mode, respectively. (h–j) As dissection occurs closer to the node, its greyness on fluorescent mode becomes a brighter and less hazy white light akin to a ‘sunrise’. (k, l) The fluorescent node elevated with a Babcock forcep seen under colour segmented fluorescence mode and overlay fluorescence mode, respectively.

Lymphatic drainage is visualized in real‐time on a display, much like during laparoscopy. The fluorescence is tracked from the injection site to the axilla (Fig. 1). SLN mapping and biopsy proceed through either the mastectomy or breast conserving surgery (BCS) incision if the SLN is within proximity. Otherwise, a separate axillary incision is made. After entering the axilla through the clavipectoral fascia, the surgical field is continuously assessed using the near‐infrared camera throughout the procedure. The fluorescent lymphatic channels are dissected and followed to the first ICG avid lymph node. The node and surrounding fat are elevated with a Babcock forcep, fat is dissected off, and its feeding artery controlled. SLN removal continues until no residual fluorescence is visible, followed by a final check for residual radioactivity with the gamma‐detecting probe. Mapping to intramammary nodes is also possible.

The near‐infrared camera offers multiple viewing conditions, including fluorescence mode, colour segmented fluorescence mode, and overlay fluorescence mode. Contrast between the SLN and background tissue is the highest under fluorescence mode. There is a “sunrise” effect as the closer the dissection is to the correct plane of the node, the fluorescence intensity increases and becomes less hazy. This is similar to a sunrise when the upper rim of the sun continues to become less hazy as it rises and appears on the horizon in the morning.

ICG fluorescence enhances SLN mapping by enabling sequential nodal dissection with real‐time visualization of lymphatics. The technique allows dynamic tracking of lymphatic drainage from the injection site to the SLN, with ICG persisting in the node for up to 60 minutes post‐injection. In contrast, BD and lymphoscintigraphy provide static feedback, as they only provide a signal where sufficient accumulation of tracer has occurred in nodes.^10^, ^11^

Adopting new surgical technologies is beneficial when they can be easily learned and integrated into existing practice. Based on the author's experience, ICG fluorescence follows a straightforward protocol and may involve a shorter learning curve compared to lymphoscintigraphy, as it appears to require less navigational skill.^12^

## Author contributions

**Chu Luan Nguyen:** Conceptualization; writing – review and editing. **Deepali Poels:** Resources; writing – review and editing. **Basilie Teoh:** Resources; writing – review and editing. **Jue‐Li Seah:** Resources; writing – review and editing. **Carlo Pulitano:** Conceptualization; resources; supervision. **Sanjay Warrier:** Conceptualization; resources; supervision.

## Supporting information


**Video 1.** Demonstration of left axillary sentinel lymph node biopsy utilizing ICG fluorescence.
Fluorescence mode: fluorescence image displayed in greyscale, providing the highest level of contrast between fluoresced and non‐fluoresced tissue; Colour segmented fluorescence mode: white light image is displayed in greyscale with fluorescence overlaid in a colour scale. Increasing intensities of fluorescence transition from blue to yellow to red; Overlay fluorescence mode: fluorescence image (green) is displayed over a 1080p white light image.
